# Assessing Gonadotropin Receptor Function by Resonance Energy Transfer-Based Assays

**DOI:** 10.3389/fendo.2015.00130

**Published:** 2015-08-27

**Authors:** Mohammed Akli Ayoub, Flavie Landomiel, Nathalie Gallay, Gwenhael Jégot, Anne Poupon, Pascale Crépieux, Eric Reiter

**Affiliations:** ^1^Biologie et Bioinformatique des Systèmes de Signalisation (BIOS) Group, INRA, UMR85, Unité Physiologie de la Reproduction et des Comportements, Nouzilly, France; ^2^CNRS, UMR7247, Nouzilly, France; ^3^Université François Rabelais, Tours, France; ^4^L’Institut français du cheval et de l’équitation (IFCE), Nouzilly, France; ^5^LE STUDIUM ^®^ Loire Valley Institute for Advanced Studies, Orléans, France

**Keywords:** gonadotropins, FSHR, LHR, GPCRs, G proteins, arrestins, BRET, FRET

## Abstract

Gonadotropin receptors belong to the super family of G protein-coupled receptors and mediate the physiological effects of follicle-stimulating hormone (FSHR) and luteinizing hormone (LHR). Their central role in the control of reproductive function has made them the focus of intensive studies. Upon binding to their cognate hormone, they trigger complex signaling and trafficking mechanisms that are tightly regulated in concentration, time, and space. Classical cellular assays often fail to capture all these dynamics. Here, we describe the use of various bioluminescence and fluorescence resonance energy transfer (BRET and FRET) assays to investigate the activation and regulation of FSHR and LHR in real-time, in living cells (i.e., transiently expressed in human embryonic kidney 293 cells). Indeed, the dynamics of hormone-mediated heterotrimeric G protein activation, cyclic adenosine-monophosphate (cAMP) production, calcium release, β-arrestin 2 recruitment, and receptor internalization/recycling was assessed. Kinetics and dose–response analyses confirmed the expected pharmacological and signaling properties of hFSHR and hLHR but revealed interesting characteristics when considering the two major pathways (cAMP and β-arrestin 2) of the two receptors assessed by BRET. Indeed, the EC_50_ values were in picomolar range for cAMP production while nanomolar range was observed for β-arrestin 2 recruitment as well as receptor internalization. Interestingly, the predicted receptor occupancy indicates that the maximal G protein activation and cAMP response occur at <10% of receptor occupancy whereas >90% of activated receptors is required to achieve full β-arrestin 2 recruitment and subsequent receptor internalization. The rapid receptor internalization was also followed by a recycling phase. Collectively, our data reveal that β-arrestin-mediated desensitization, internalization, and the subsequent fast recycling of receptors at the plasma membrane may provide a mechanistic ground to the “spare receptor” paradigm. More generally, the novel tools described here will undoubtedly provide the scientific community investigating gonadotropin receptors with powerful means to decipher their pharmacology and signaling with the prospect of pathophysiological and drug discovery applications.

## Introduction

The gonadotropin receptors play a central role in the control of mammal reproduction by mediating the physiological responses of the two major pituitary glycoprotein hormones, follicle-stimulating hormone (FSH) and luteinizing hormone (LH). Their respective receptors, follicle-stimulating hormone receptor (FSHR) and luteinizing hormone receptor (LHR), are mainly expressed in the gonads where they control the ovarian and testicular functions in females and males, respectively, by regulating both steroidogenesis and gametogenesis (1, 2). Both FSHR and LHR belong to a subgroup of class A (rhodopsin-like) G protein-coupled receptors (GPCRs) characterized by the presence of multiple leucine-rich repeats (LRRs) in their extracellular amino-terminal domain. This subgroup also includes the thyroid-stimulating hormone receptor (TSHR) and the receptors for the peptidic hormone relaxin and INSL3 (RXFP1 and 2). The LRRs containing region in FSHR has been shown to be determinant for its interaction with FSH (3–5). In terms of the intracellular signaling, FSHR and LHR are known to mediate the canonical G protein-mediated signaling pathway through coupling to heterotrimeric Gαs proteins, which activates the adenylate cyclase, resulting in an increase in intracellular cyclic adenosine-monophosphate (cAMP) levels and activation of protein kinase A (PKA) as well as the exchange protein directly activated by cAMP (EPAC). This in turn triggers the activation of multiple downstream kinases that modulate the nuclear activity of cAMP response element-binding protein (CREB) and the expression of the genes involved in the physiological responses of the gonadotropins. However, recent evidences point to a multiplicity of the signaling that can be mediated by FSHR and LHR by engaging additional G protein-dependent and independent pathways [for review, see Ref. (6–9)], including β-arrestin-dependent pathways (10–13). As a consequence, similar to most other GPCRs, these receptors’ pharmacology and signaling involve highly diverse and complex mechanisms. Therefore, the use of recent innovative technologies to investigate these receptors could certainly help understanding better their activation mode.

Among the emerging methods to study GPCRs, the focus is on energy transfer-based assays that rely on the biophysical bioluminescence and fluorescence resonance energy transfer (BRET and FRET) technologies. These approaches link the concept of distance/proximity, in space and time, between an energy donor and an energy acceptor to the biological question of interest according to Förster’s Law in both static and dynamic configurations (14–16). Since their development, BRET and FRET have been extensively used to study different cellular and molecular aspects related to the function and regulation of cell surface receptors, such as GPCRs and tyrosine kinase receptors (TKRs) (17, 18). In fact, GPCRs constitute the research field of choice where BRET/FRET are elegantly used and are being the subject of permanent development and improvement (16, 19–21). Indeed, by using BRET and FRET, it is now possible to quantitatively address, in real-time and live cells, different questions about the functioning of GPCRs including ligand binding, receptor activation, G protein coupling, intracellular downstream signaling, β-arrestin recruitment, receptor trafficking, and oligomerization (16, 19–26). In this context, BRET and FRET significantly contributed to major recent advances in the field with the emergence of new concepts, such as receptor heteromerization, receptor/G protein preassembly, and biased signaling. Even though these advances further illustrate the complexity of the GPCR functioning, they pushed the scientific community one step further in understanding better the involvement of GPCRs in physiology and pathophysiology. However, the application of BRET and FRET approaches to the gonadotropin receptors has remained limited to date. In this study, we report the application of a series of novel BRET and FRET assays to study the activation and regulation of the human gonadotropin receptors, hFSHR and hLHR/hCGR (here designed as hLHR), when they are transiently expressed in HEK 293 cells. Kinetics and dose–response analyses using various assays were performed in 96- and 384-well formats in real-time and live cells.

## Materials and Methods

### Materials and plasmid constructions

The plasmid encoding human FSHR was generated as previously described (12). The other plasmids encoding the different BRET/FRET sensors and fusion proteins were generously provided as follows: hLHR from A. Ulloa-Aguirre (Universidad Nacional Autónoma de México, México, Mexico), different Rluc8- and Venus-fused G proteins from J. P. Pin (Functional Genomics Institute, Montpellier, France) and K. D. Pfleger (Harry Perkins Institute of Medical Research, Perth, WA, Australia) (also hV2R–Rluc8), Rluc8-fused hFSHR and hLHR from A. Hanyaloglu (Imperial College, London, UK), yPET-β-arrestin 2 from M. G. Scott (Cochin Institute, Paris, France), Aequorin-GFP from B. Lambolez (Pierre et Marie Curie University, Paris, France), CAMYEL from L. I. Jiang (University of Texas, TX, USA), ICUE from J. Zhang (The Johns Hopkins University, Baltimore, MD, USA), and Venus-KRas from N. A. Lambert (Georgia Health Sciences University, Augusta, GA, USA). Recombinant hFSH was kindly gifted by Merck-Serono (Darmstadt, Germany), hCG was kindly donated by Y. Combarnous (CNRS, Nouzilly, France), forskolin and DDAVP were purchased from Sigma-Aldrich (St. Louis, MO, USA). All the 96- and 384-well white microplates were from Greiner Bio-One (Courtaboeuf, France). Coelenterazine h substrate was purchased from Interchim (Montluçon, France).

### Cell culture and transfection

HEK 293 cells were grown in complete medium (DMEM supplemented with 10% (v/v) fetal bovine serum, 4.5 g/l glucose, 100 U/ml penicillin, 0.1 mg/ml streptomycin, and 1 mM glutamine) (all from Invitrogen, Carlsbad, CA, USA). Transient transfections were performed by reverse transfection in 96-well plates using Metafectene PRO (Biontex, München, Germany) following the manufacturer’s protocol. Briefly, for each well, the different combinations of coding plasmids were used as follows: 200 ng of total plasmid per well were resuspended in 25 μl of serum-free DMEM and mixed with Metafectene PRO (0.5 μl/well) previously preincubated 5 min at room temperature in 25 μl serum-free DMEM (2 × 25 μl/well). Then the two solutions of serum-free DMEM-containing plasmids and Metafectene PRO were mixed and incubated for 20 min at room temperature. Cells (10^5^ in 200 μl/well) in DMEM supplemented with 10% FCS were then incubated with the final plasmid-Metafectene PRO mix (50 μl/well). Transfection efficiency was typically in the 60–70% range and the correct expression of the different fusion proteins used for BRET and FRET was examined by fluorescence and luminescence measurements using a Mithras LB 943 plate reader (Berthold Technologies GmbH and Co. Wildbad, Germany).

### BRET measurements

Forty-eight hours after transfection, cells were washed with PBS and BRET measurements were performed depending on the experiments as described previously (27). For the endpoint dose–response analysis, cells were first preincubated 30 min at 37°C in 40 μl/well of PBS 1×, HEPES 5 mM, 200 μM IBMX (for cAMP assays) containing or not increasing concentrations of hFSH or hCG as indicated. Then BRET measurements were performed upon addition of 10 μl/well of coelenterazine h (5 μM final) using a Mithras LB 943 plate reader. For the real-time BRET kinetics, cells were first resuspended in 60 μl/well of PBS-HEPES 10 mM (+IBMX 200 μM for cAMP assays) and then BRET measurements were immediately performed upon addition of 10 μl/well of coelenterazine h (5 μM final) and 10 μl/well of the sub-maximal concentrations of hFSH or hCG (fivefold concentrated).

### Calcium measurements using aequorin-GFP

Forty-eight hours after transfection, cells co-expressing hFSHR and aequorin-GFP (AEQ-GFP) were incubated for 3 h with 40 μl/well of coelenterazine h substrate (5 μM final) in PBS 1×, HEPES 10 mM, BSA 0.1%, in the dark, and at 37°C to allow aequorin reconstitution. Luminescence emission at 480 and 540 nm was then measured in each well individually every 0.05 s before and after the rapid injection of 10 μl/well of hFSH (fivefold concentrated) or of vehicle, using the injection system and the dual emission detection of a Mithras LB 943 plate reader.

### cAMP accumulation measured by HTRF^®^

Intracellular cAMP levels were measured using a homogeneous time-resolved fluorescence (HTRF^®^) cAMP dynamic 2 assay kit (CisBio Bioassays, Bagnol sur Cèze, France) (28). Forty-eight hours post-transfection cells were detached and seeded into white 384-well microplates with 5,000 cells/well in 5 μl of stimulation buffer (PBS 1×, 200 μM IBMX, 5 mM HEPES, 0.1% BSA). For their stimulation, 5 μl/well of the stimulation buffer containing or not different doses of hFSH and hCG as indicated were added. Then, cells were incubated for 30 min at 37°C and then lysed by addition of 10 μl/well of the supplied conjugate-lysis buffer containing d2-labeled cAMP and Europium cryptate-labeled anti-cAMP antibody, both reconstituted according to the manufacturer’s instructions. Plates were incubated for 1 h in the dark at room temperature and time-resolved fluorescence signals were measured at 620 and 665 nm, respectively, 50 ms after excitation at 320 nm using a Mithras LB 943 plate reader.

### cAMP accumulation measured by microscopic FRET assay

Forty-eight hours after transfection, cells co-expressing the cAMP sensor (ICUE) with either hFSHR or hLHR were plated in imaging dishes and imaged in the dark at 37°C on a temperature-controlled stage using a Leica DM IRB (Leica Microsystems) microscope with a CoolSnap fx cooled charge-coupled device camera (Ropper Scientific) controlled by METAFLUOR 7.5 (Universal Imaging Corporation, Downingtown, PA, USA). Dual emission ratio imaging was carried out using a 436DF10 excitation filter, a 436–510 DBDR dichroic mirror, and 480-AF30 and 550-AF30 emission filters for CFP and YFP, respectively. Exposure time was 400 ms and images were taken every 30 s. Typically, equal sensor-positive cells and non-specific areas were chosen in the field of the microscope. The evolution of fluorescence was recorded individually in each area for the whole duration of the experiments. Several independent plates were analyzed according to this procedure, and the specific FRET signal of each cell (positive minus negative area) was pooled. Cells displaying a whole range of intensities were selected and analyzed without any impact of the expression level of the sensor on the responsiveness being noticed. After 5 min of baseline measurement, cells were stimulated with either 1 nM of hFSH or hCG, and 1 μM of forskolin was added as a positive control after 20 min of stimulation. A low hormone dose has been chosen in order to avoid saturation of the ICUE sensor, which has a limited dynamic range compared to BRET assays. Fluorescent intensity of non-specific areas was subtracted to the intensity of fluorescent cells expressing the sensor in order to quantify the specific signal. The FRET ratio (CFP/YFP) was calculated for each individual cell. Data represent the mean ± SEM of at least 20 individual cell responses measured in three independent experiments.

### β-arrestin recruitment assessed by TANGO assay

This assay was carried out as previously described by Barnea et al. (29). We generated HTLA cells (HEK293T-derived cell line containing a stably integrated tTA-dependent firefly luciferase reporter gene) stably expressing FSHR/AVPR2-CT chimera, and β-arrestin 2-TEV fusion protein. Growing HTLA hFSHR cells were plated in white 96-well assay plates at 4 × 10^4^ cells per well in MEM, supplemented with 10% FBS, glutamine, and antibiotic cocktail. Twenty-four hours after plating, increasing concentrations of hFSH were added and cells were cultured for 14–20 h before measuring reporter gene activity. Luciferase activity was determined by using the Bright-Glo luciferase assay system (Promega, Charbonnieres, France), following the manufacturer’s protocol, and using a POLARstar OPTIMA luminometer (BMG Labtech, Ortenberg, Germany).

### Data analysis

Bioluminescence resonance energy transfer data are represented either as 480 nm/540 nm (ICUE sensor), 540 nm/480 nm (β-arrestin and internalization kinetics), or as hFSH/hCG-induced BRET changes by subtracting the ratio 540 nm/480 nm of luminescence in a well of PBS-treated cells from the same ratio in wells where the cells were treated with hFSH or hCG. In this calculation, only ligand-induced BRET changes (increase or decrease) are represented and the PBS-treated cell sample represents the background eliminating the requirement for measuring an Rluc-only control sample since fast kinetics and dose–response analyses were performed. Kinetic and dose–response curves were fitted following the appropriate non-linear regression equations using Prism GraphPad software (San Diego, CA, USA). Statistical analyses were performed using two-way ANOVA included in Prism GraphPad software.

## Results

### Receptor-mediated cAMP production

**Figure 1:**
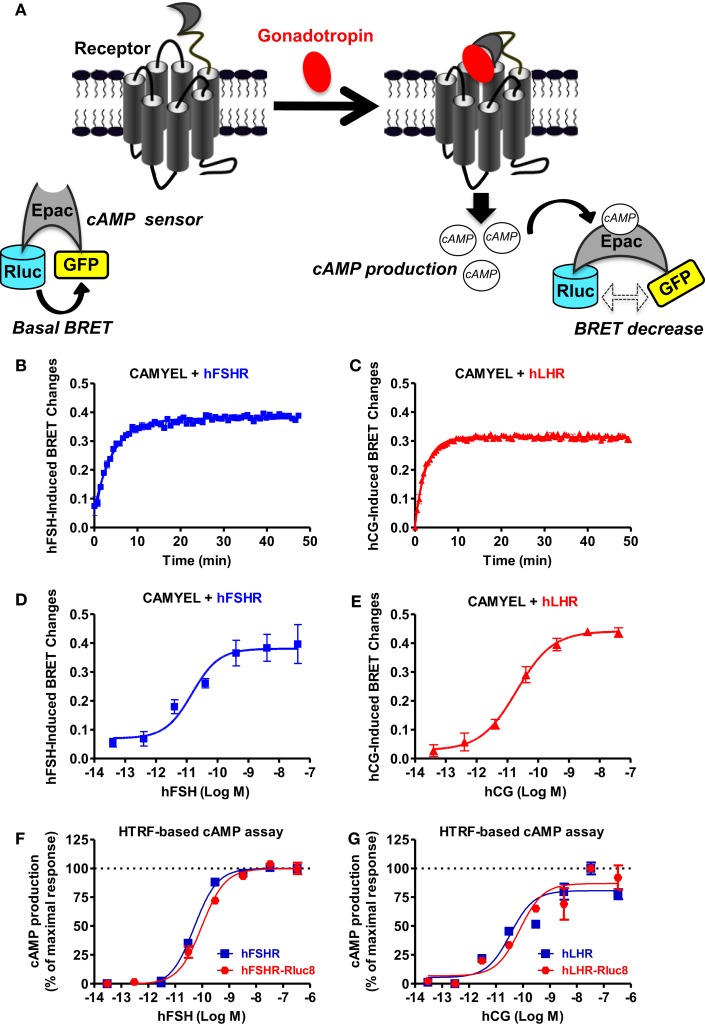
**BRET- and HTRF-based cAMP production assays**. **(A)** Principle of the BRET-based cAMP assay (CAMYEL sensor). HEK 293 cells transiently expressing the indicated proteins (wildtype receptors and CAMYEL for BRET; wildtype or Rluc8-tagged receptors for HTRF) were stimulated with either 5 nM (for kinetics) or increasing concentrations (for dose–response curves) of hFSH **(B,D,F)** or hCG **(C,E,G)** as indicated. Then BRET and HTRF measurements format were performed as described in the Section “Materials and Methods” in 96-well and 384-well plates, respectively. Data are means ± SEM of three experiments performed either in a single point or triplicate.

First, we examined cAMP response by hFSHR and hLHR since they are both known to couple to heterotrimeric Gs protein, leading to adenylyl cyclase activation and ultimately to an increase in the intracellular cAMP levels. To this end, we used the BRET-based cAMP sensor, CAMYEL, developed by Jiang et al. (30) (Figure 1A), which allows the assessment of intracellular cAMP changes in real-time and live cells. Under basal conditions, a high-BRET signal occurs between the donor (*Renilla* luciferase or Rluc) and the acceptor (green fluorescent protein or GFP) due to the favorable conformation and proximity/orientation of the Rluc and GFP within the Epac motif composing the sensor. In contrast, an increase in the cytosolic cAMP concentrations and its binding to Epac induce changes in the conformation of Rluc-Epac-GFP sensor, resulting in a significant decrease in the BRET signal (Figure 1A). The receptors were transiently co-expressed with CAMYEL in HEK 293 cells and real-time kinetics were conducted (Figures 1B,C) at different doses allowing the inference of sigmoidal dose–response curves (Figures 1D,E). Kinetic analyses showed a relatively fast cAMP response (*t*_1/2_ of 3.2 and 1.7 min for FSHR and LHR, respectively) (Table 1) upon stimulation with 5 nM of gonadotropins with a plateau reached after ~10 min for both hFSHR (Figure 1B) and hLHR (Figure 1C). As expected, both hormones, hFSH (Figure 1D) and hCG (Figure 1E), showed very potent effects on their specific receptors with EC_50_ values in the picomolar range (Table 1). Similar results were obtained using HTRF^®^-based cAMP assay (28) on both hFSHR (Figure 1F) and hLHR (Figure 1G) either wildtype or Rluc8-fused receptors. This indicates that both Rluc8-fused receptors retained correct expression and function and can therefore be used in BRET assays for the recruitment of β-arrestin 2 and receptor internalization (Figures 5 and 6).

**Table 1:** **EC_50_ and *t*_1/2_ values for gonadotropin-promoted cAMP production, β-arrestin 2 recruitment and receptor internalization/recycling of hFSHR and hLHR**.

Receptors	cAMP production			BRET G proteins	β-arrestin 2 recruitment			Internalization	Recycling
	
	EC_50_ (pM) BRET	EC_50_ (pM) HTRF	*t*_1/2_ (min) BRET	*t*_1/2_ (s) BRET	EC_50_ (nM) BRET	EC_50_ (nM) TANGO	*t*_1/2_ (min) BRET	EC_50_ (nM) BRET	*t*_1/2_ (min) BRET
hFSHR	3.0 ± 1.2	1.4 ± 0.1	3.2 ± 0.3	16.8 ± 11.7^a^ 10.9 ± 0.3^b^	3.7 ± 2.0*	5.7 ± 2.6*	4.8 ± 0.3	2.6 ± 1.0*	10.5 ± 0.2
hLHR	3.5 ± 2.2	1.9 ± 0.3	1.7 ± 0.1	ND	2.0 ± 0.1	ND	6.6 ± 0.2	4.9 ± 1.9	8.6 ± 0.3

*Data are means ± SEM of three to four independent experiments*.

*^a^BRET between Gαs-Rluc8 and Venus-Gγ2*.

^b^BRET between Gαs-Rluc8 and Venus-Gβ1, both calculated by fitting the curves in Figures 3B,D using “plateau then one phase decay equation.”

* < 0.05 versus EC values measured in cAMP production assay.

**Figure 2:**
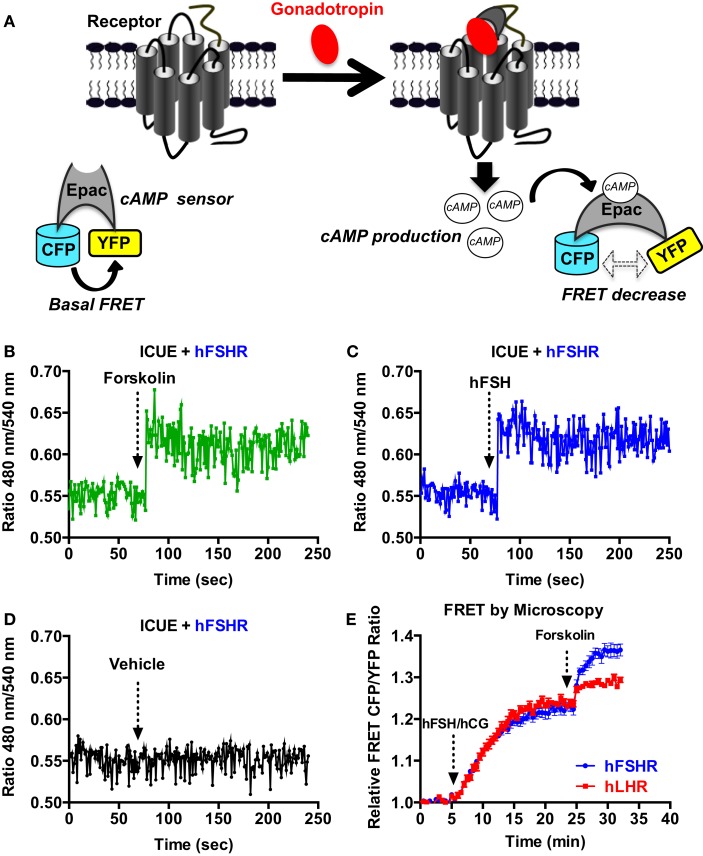
**FRET-based cAMP production assays**. **(A)** Principle of the FRET-based cAMP assay (ICUE sensor). HEK 293 cells transiently co-expressing ICUE sensor with either hFSHR or hLHR **(E)** were used for FRET measurements using either 96-well format **(B–D)** or microscopy on individual cells **(E)**. Cells were stimulated with either 1 μM of forskolin **(B,E)**, 5 nM of hFSH **(C)**, 1 nM of hFSH or hCG **(E)**, or vehicle **(D)** as indicated. Then FRET measurements were performed as described in the Section “Materials and Methods.” Data are representative of three independent experiments performed in single points **(B–D)** or 16 individual cells **(E)**.

We also used a FRET-based cAMP sensor (ICUE) allowing real-time measurements of cAMP production as previously shown (31) (Figure 2A) using both real-time FRET measurements in 96-well plate format every 0.5 s as well as individual cell analysis with the appropriate fluorescence microscopy setting. The 96-well plate format clearly allowed to measure very rapid changes in the FRET signals in cells co-expressing ICUE and hFSHR and challenged with 1 μM of forskolin (Figure 2B) or 5 nM of hFSH (Figure 2C). These changes were specific to hFSH/forskolin-induced cAMP production since vehicle injection did not induce any change in the FRET signal (Figure 2D). In parallel, the FRET analysis by fluorescence microscopy on individual stimulated cells co-expressing ICUE and either hFSHR or hLHR showed a time-dependent increase of cAMP production induced by 1 nM of hFSH or hCG, respectively, as well as 1 μM of forskolin (Figure 2E), as previously reported (12).

### Receptor-Gαs protein coupling assessed by BRET

**Figure 3:**
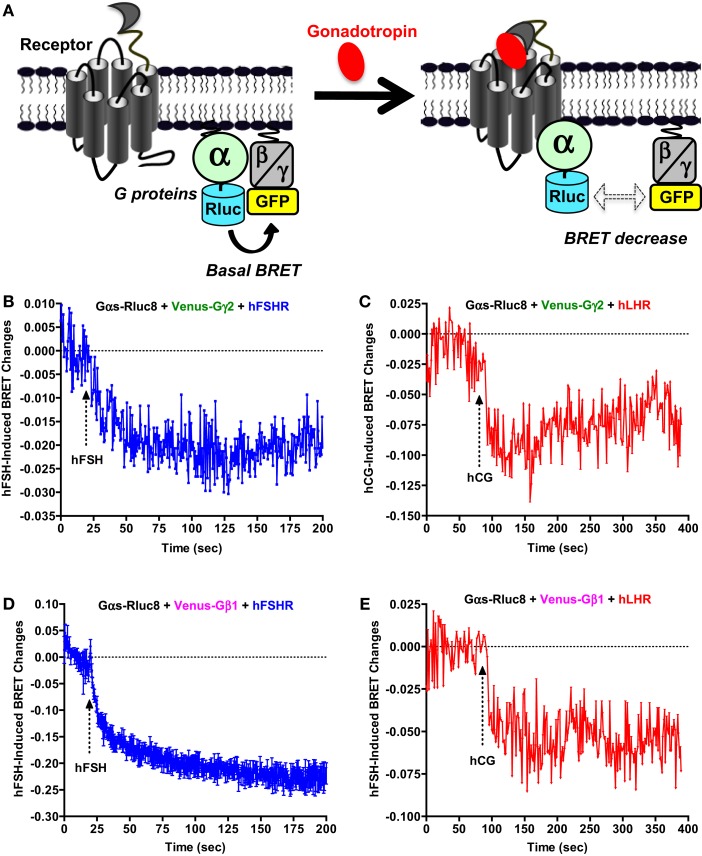
**G protein activation assessed by BRET**. **(A)** Principle of the BRET-based G protein assay. HEK 293 cells transiently co-expressing the indicated Rluc8- and Venus-fused G protein subunits with either hFSHR **(B,D)** or hLHR **(C,E)** were used for BRET measurements using 96-well format performed before and after injection of 10 nM hFSH or hCG as described in the Section “Materials and Methods.” Data are representative of three independent experiments performed in single points.

Next, we examined the functional coupling of hFSHR and hLHR to the heterotrimeric G protein (Gαs and Gβγ) in real-time and live cells by measuring BRET changes between the different G protein subunits as previously reported (27, 32–36). In this assay, a change (in this case a decrease) in the proximity/association between the Gα subunit and Gβγ dimer as well as their conformation upon receptor activation is assessed in time-dependent manner reflecting the functional coupling of the receptor with its cognate heterotrimeric G protein (Figure 3A). Gαs-Rluc8 was transiently co-expressed with either Venus-Gγ2 (Figures 3B,C) or Venus-Gβ1 (Figures 3D,E) in the presence of hFSHR or hLHR as indicated. BRET changes were then rapidly assessed every 0.5 s before and after receptor activation by the injection of 10 nM of hFSH or hCG. As shown, hFSH nicely induced a very rapid and significant BRET decrease between Gαs-Rluc8 and Venus-Gγ2 (Figure 3B) and Venus-Gβ1 (Figure 3D) co-expressed with hFSHR. Similar albeit noisier effects were observed with 10 nM of hCG on BRET between Gαs-Rluc8 and Venus-Gγ2 (Figure 3C) and Venus-Gβ1 (Figure 3E) in the context of hLHR expressing cells. Such BRET changes likely reflect the activation of Gαs protein by the hFSHR and hLHR and are consistent with the cAMP measurements shown in Figures 1 and 2. Our data are consistent with the previous BRET data reported for other GPCRs, showing a decrease of the BRET signals between Gαs and Gβ/γ subunits (32, 33, 35, 36). As expected, the observed kinetics with these sensors was much faster than the one measured for cAMP (i.e., *t*_1/2_ between 10 and 16 s) (Table 1).

### Receptor-mediated calcium release

**Figure 4:**
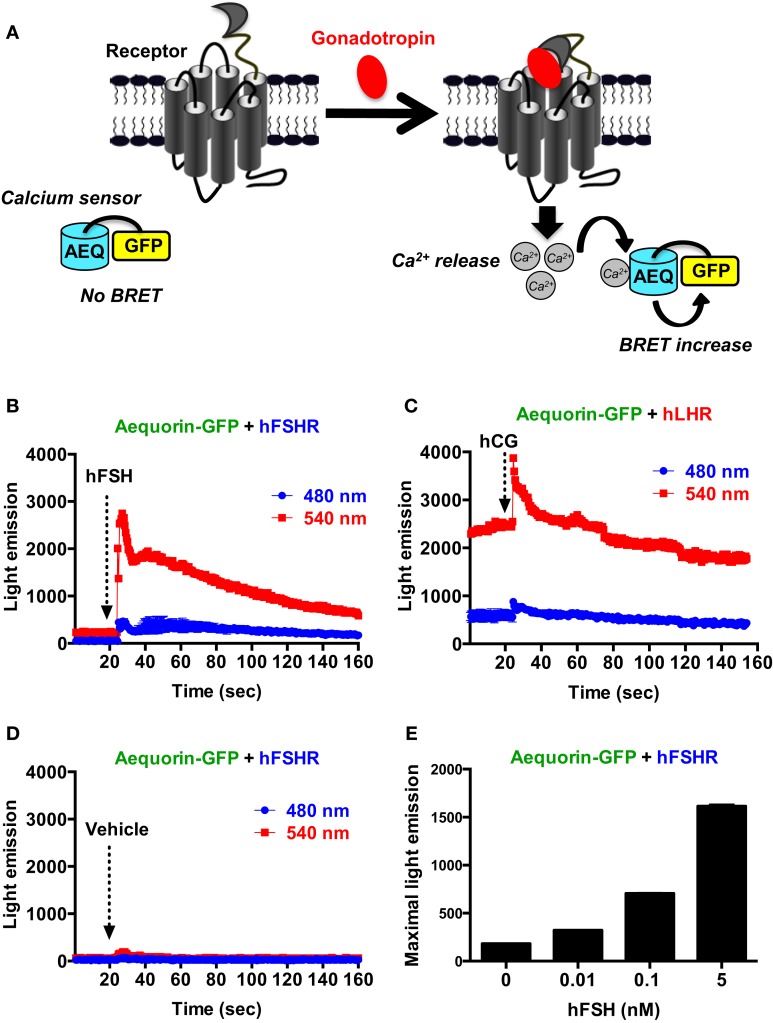
**BRET-based calcium assay**. **(A)** Principle of the BRET-based calcium assay. HEK 293 cells transiently co-expressing the AEQ-GFP sensor with either hFSHR **(B,D,E)** or hLHR **(C)** were used for light emission measurements using 96-well format before and after injection of vehicle **(D)** 10 nM hFSH or hCG or various doses of hFSH **(E)** as described in the Section “Materials and Methods.” Data are representative of three independent experiments performed in single points.

We also assessed the intracellular calcium release mediated by the activation of gonadotropin receptors as previously reported (37, 38). For this, we used an aequorin-dependent calcium assay (AEQ-GFP) based on luminescence and BRET increase upon binding of calcium to the aequorin protein fused to GFP (39, 40) (Figure 4A). In the presence of calcium, aequorin emits luminescence at 480 nm part of which is transferred to GFP due to their sufficient proximity leading to GFP excitation and light emission at 540 nm. As shown in Figure 4B, in cells co-expressing AEQ-GFP and hFSHR, a significant and rapid increase in light emission at 540 nm occurred upon cell stimulation with 10 nM of hFSH (Figure 4B) indicating intracellular calcium increase. However, in cells co-expressing AEQ-GFP and hLHR, a significant basal emission at 540 nm was observed and stimulation with 10 nM hCG only induced weaker response (Figure 4C) as compared to hFSH on its receptor (Figure 4B). Such an effect was specific to gonadotropins since no increase in light emission was observed in AEQ-GFP and hFSHR co-expressing cells upon vehicle injection (Figure 4D) and the hFSH-promoted luminescence increase was dose-dependent (Figure 4E). Moreover, no significant light emission was measured in cells expressing AEQ-GFP alone and stimulated with 10 nM of hFSH (data not shown).

### Recruitment of β-arrestin 2 assessed by BRET

**Figure 5:**
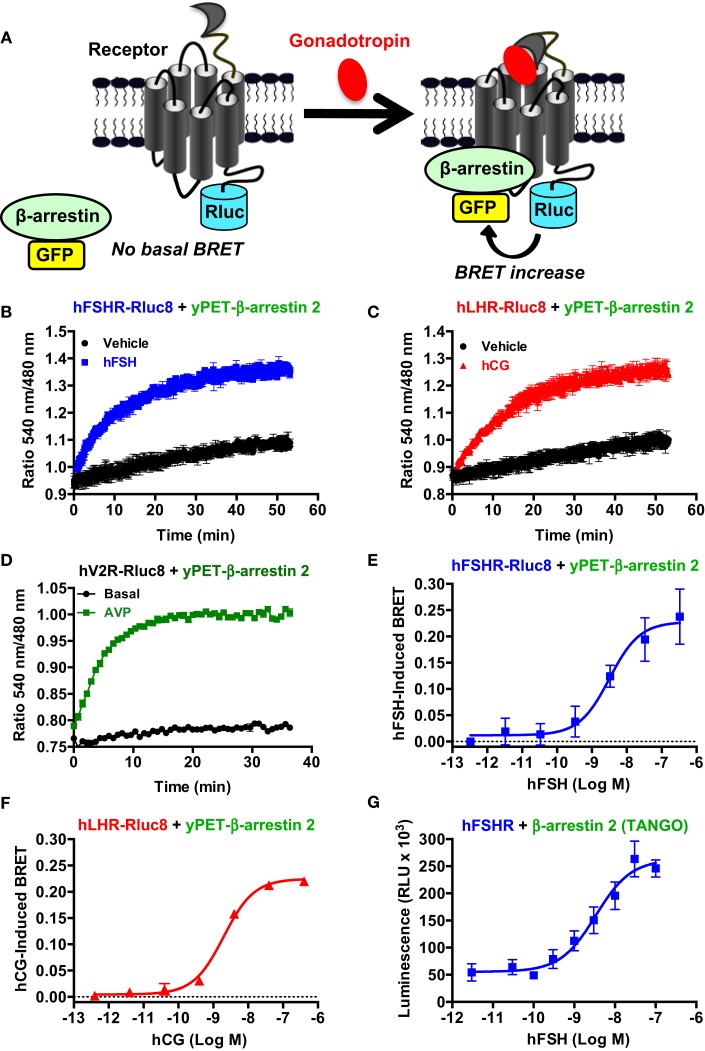
**β-arrestin 2 recruitment assessed by BRET**. **(A)**. Principle of the BRET-based β-arrestin assay. HEK 293 cells transiently co-expressing yPET-β-arrestin 2 with either hFSHR-Rluc8 **(B,E,G)**, hLHR-Rluc8 **(C,F)** or hV2R–Rluc8 **(D)** were used for BRET measurements in 96-well format using both real-time kinetics under basal (vehicle) and stimulated conditions in the presence of 10 nM of hormones **(B,C)** and endpoint signal recording after 30 min of stimulation with increasing hormone concentrations **(D–F)** as described in the Section “Materials and Methods.” In parallel, hFSHR/β-arrestin 2 association was also assessed in dose-dependent way using TANGO assay in 384-well format **(G)**. Data are means ± SEM of three to four independent experiments performed in triplicate points.

The role of β-arrestins not only in desensitization/internalization but also in signaling of GPCRs is now well established (41–43) and this has been previously reported for the FSHR (10, 11, 13, 44) and LHR (45). Here, we examined for the first time the recruitment of β-arrestins to activated hFSHR and hLHR in real-time and live cells using BRET technology as illustrated in Figure 5A. Indeed, under the inactive conformation of the receptors, β-arrestins are mostly cytosolic. Upon activation, the receptors are phosphorylated by G protein-coupled receptor kinases (GRKs) leading to the translocation of β-arrestins from the cytosol to the intracellular domains of the receptors triggering their desensitization, internalization, and signaling. The BRET increase between the receptors and β-arrestins is used to assess this process in real-time and living cells. For this purpose, the receptors were fused to BRET donor (Receptor-Rluc8) and co-expressed with β-arrestin 2 fused to BRET acceptor (here yPET as a GFP variant) and the translocation of β-arrestin 2 to the receptor was then measured before and after receptor activation (Figure 5A). The functionality of FSHR-Rluc8 and hLHR-Rluc8 is verified by the cAMP assay shown in Figures 1F,G. Dose–response and kinetics experiments were carried out in cells co-expressing yPET-β-arrestin 2 and either hFSHR-Rluc8 (Figures 5B,E) or hLHR-Rluc8 (Figures 5C,F). Real-time kinetic analyses showed a significant BRET increase over the basal signal with hFSHR-Rluc8 (Figure 5B) or hLHR-Rluc8 (Figure 5C) and yPET-β-arrestin 2 upon stimulation with 10 nM of hFSH or hCG, respectively. The BRET increase occurred in a time-dependent manner with a sustained plateau reached after 20–30 min of receptor activation indicating a class B profile according to the common GPCR classification with respect to β-arrestin association (46). We used the human vasopressin V2 receptor (hV2R–Rluc8) as a prototype for class B GPCR in our BRET assay and observed a similar kinetic profile compared to hFSHR and hLHR (Figure 5D). Moreover, the effects were dose-dependent for both hFSH (Figure 5E) and hCG (Figure 5F) on their respective receptors with EC_50_ values largely higher (i.e., nanomolar range) than those observed for cAMP signaling (Figure 1; Table 1). Such shift in the hormone potencies is not due to the effect of fusion of the receptors with Rluc8 since these constructs showed cAMP responses similar to that observed with their corresponding wild type receptors (Figures 1F,G). Moreover, dose–response experiments were also performed using an indirect TANGO assay on hFSHR bearing the vasopressin receptor 2 (V2R) C-terminus and showed similar potency of hFSH on hFSHR/β-arrestin 2 association as assessed by BRET (Figure 5G).

### Receptor internalization and recycling assessed by BRET

**Figure 6:**
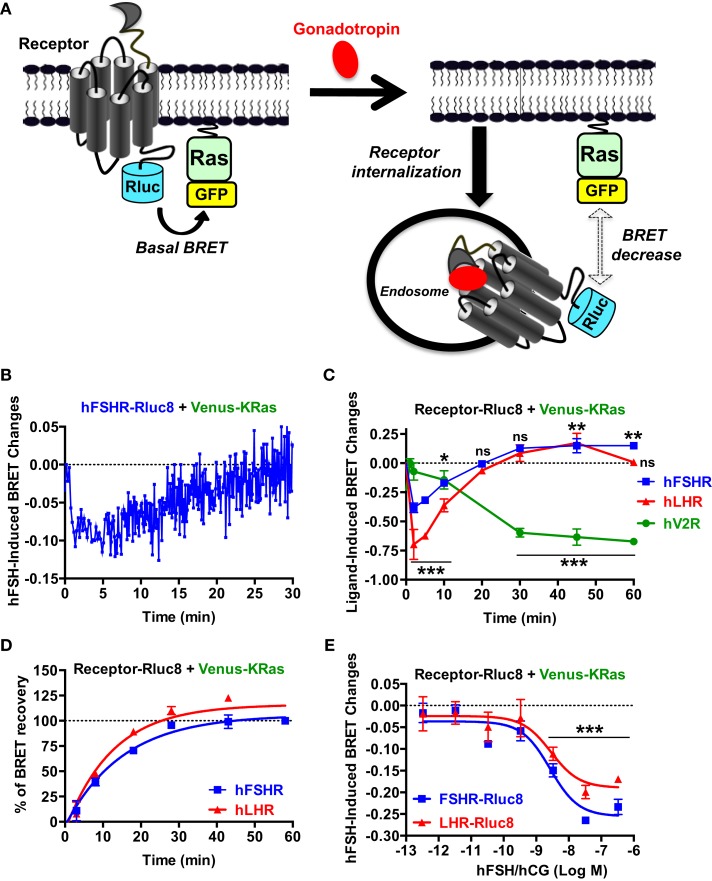
**Receptor internalization and recycling assessed by BRET**. **(A)** Principle of the BRET-based receptor internalization assay. HEK 293 cells transiently co-expressing Venus-KRas with either hFSHR-Rluc8, hLHR-Rluc8, or hV2R–Rluc8 were used for BRET measurements using 96-well format as described the Section “Materials and Methods.” Real-time kinetics **(B)** and dose–response analysis **(E)** after 2 min of stimulation with 10 nM **(B)** or increasing concentrations **(E)** of the hormones. In addition, time-course experiments were performed upon cell stimulation with 10 nM of the indicated agonists for 2, 5, 10, 20, 30, 45, and 60 min **(C)**. The BRET recovery phases for hFSHR and hLHR in **(C)** were also fitted using a non-linear regression (one phase kinetic equation) and by taking the signals after 2 and 60 min as 0 and 100% of recovery, respectively **(D)**. This allowed the calculation of *t*_1/2_ values of receptor recycling indicated in Table 1. Data are means ± SEM of three independent experiments performed in triplicate points. *p < 0.05, **p < 0.01, ***p < 0.001, ns, not significant compared to unstimulated controls.

Finally, we examined gonadotropin-induced receptor internalization in real-time and live cells using BRET between the Rluc8-tagged receptors (BRET donor) and a plasma membrane marker, KRas, fused to BRET acceptor (Venus, another GFP variant), as recently described (47). This assay is based on the changes in the physical proximity between KRas and the receptors at the plasma membrane upon receptor activation and thereby internalization as illustrated in Figure 6A. The agonist-induced decrease in the high basal BRET signals was assessed in cells co-expressing Venus-KRas with either hFSHR-Rluc8 or hLHR-Rluc8 (Figure 6A). We observed a very rapid decrease in the BRET signal between hFHSR-Rluc8 and Venus-KRas following cell stimulation with 10 nM of hFSH to reach the maximal decrease up to 2–5 min post-stimulation, indicating the rapid internalization of hFSHR under our conditions (Figure 6B). Interestingly, we observed a recovery phase of the BRET signal after 5–10 min of stimulation, which returns back to the basal level after 20 min suggesting recycling of the internalized receptors (Figure 6B). To confirm this observation on both hFSHR and hLHR, we performed time-course analysis where cells were first preincubated with hFSH or hCG at different times at 37°C before BRET signals were measured. The BRET measurements showed a maximal internalization of both receptors after 2–5 min and a total recovery of the BRET signals after 20 min (Figure 6C). Interestingly, the recovery phase continued to increase after 30 min to reach maximal BRET signals even higher than the basal levels after 45–60 min (Figure 6C), suggesting the recycling of the internalized receptors and/or the recruitment of an intracellular pool of receptors. Such behavior was specific to hFSHR and hLHR, since it was not observed for the human vasopressin 2 receptor (hV2R–Rluc8) activated with 1 μM of AVP (Figure 6C). In fact, these data are consistent with a delayed internalization (maximum after 30 min) and absence of recycling to the plasma membrane after internalization as it is well documented for V2R (48–50). Moreover, in order to estimate the kinetic parameter of the receptor recycling, we normalized the part of the curves corresponding to the recovery phase of hFSHR and hLHR shown in Figure 6C by taking 0 and 100% of the maximal BRET changes measured after 2 (maximal internalization) and 60 min of stimulation (maximal recycling), respectively (Figure 6D). As a result, both receptors recycled with similar kinetics with a half-time of about 10 min (Table 1), indicating that the recycling of hFSHR and hLHR was slower than their internalization, at least in our system. Finally, we performed BRET dose–response experiments after 5 min of stimulation showing the decrease in the BRET signals between Rluc8-tagged receptors and Venus-KRas in a dose-dependent manner with no significant differences between the two receptors (Figure 6E). It is worth noting that the potencies of hFSH and hCG on receptor internalization were similar to that observed for the recruitment of β-arrestin 2 (Figures 5D,E; Table 1), consistent with the notion that both events may be linked.

## Discussion

In this study, we provide new insights on the activation and regulation of gonadotropin receptors by applying energy transfer-based technologies (BRET and FRET). These aspects were studied in real-time in live HEK 293 cells in dose- and time-dependent manners using various BRET configurations and BRET/FRET sensors. This allowed us to cover critical steps in the signaling of hFSHR and hLHR going from their intimate coupling to the heterotrimeric Gαs/Gβγ proteins at the membrane to the accumulation of cytosolic cAMP and calcium as well as β-arrestin 2 recruitment, receptor internalization, and recycling. Together, our data illustrate the robustness of the different BRET and FRET assays used to examine such components of GPCR activation and signaling with exquisite precision. In our hand, FRET, which gives beautiful results in fluorescence microscopy, is less suited to multiwell plate measurements than BRET since it displayed highly reduced amplitude of response. However, FRET sensors combined with microscopy offers the advantage of measuring individual cell responses.

The set of data presented in this study on hFSHR and hLHR confirm and expand previous reports from the literature using conventional approaches in terms of G protein-dependent signaling, β-arrestin recruitment, and receptor trafficking. Indeed, a high potency (picomolar range) (Table 1) was classically observed for both hFSH and hCG with respect to the activation of the canonical Gs/cAMP signaling pathway, which is thought to account for the most physiological responses of FSH and LH in the gonads, hence in the control of reproduction (1, 2, 8). BRET measurements between the Gαs and Gβγ subunits activated by hFSHR and hLHR showed relatively rapid BRET changes upon receptor activation consistent with previous observations using similar BRET assays on different heterotrimeric G proteins and GPCRs (32, 33, 35, 36, 51). Moreover, BRET-based calcium sensor allowed the assessment of rapid and transient calcium release in response to the hormones confirming previous reports of FSHR- and LHR-mediated calcium response (37, 38). Moreover, our data suggest differences between the two receptors in terms of the basal calcium response, and further investigation will be needed to better understand this aspect of FSHR/LHR signaling. One possible explanation could be that the higher basal level observed in LHR-transfected cells is a reflexion of the fact that this receptor leads to significant constitutive activity while FSHR does not (52, 53).

**Figure 7:**
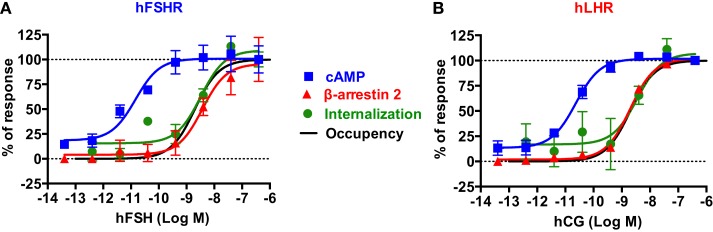
**Comparative analysis of the dose–response analysis on cAMP responses, β-arrestin 2 recruitment, and receptor internalization**. For both hFSHR **(A)** and hLHR **(B)**, the individual dose–response data obtained in each BRET assay were normalized to the maximal signal taken as 100% of receptor-mediated responses. Moreover, receptor occupancy curves were incorporated to correlate the different specific responses with the percentage of occupied receptor. The occupancy was estimated using the following equation: % Occupancy = (Ligand)/(Ligand) + *K*_d_, considering a *K*_d_ of 2 nM for both hFSH and hCG determined by radioligand binding assay on hFSHR expressed in HEK 293 cells (data not shown).

Interestingly, our BRET data provide the first direct evidence for the dynamics of receptor/β-arrestin association, in real-time and live cells, in response to FSH and hCG stimulation. We confirmed the accuracy of the measurements for hFSHR/β-arrestin association using an indirect TANGO assay, both data sets being also consistent with those recently reported on FSHR using the PathHunter β-arrestin assay from DiscoverRx (4, 5). This commercial assay, similar to our home-made TANGO assay, precludes real-time measurements since 90 min of agonist stimulation are followed by 1 h of incubation before the assay detection (overnight incubation in the TANGO assay). In addition, it is worth noting that non-trivial modifications are introduced in the C-terminus of FSHR in these two assays, although this region is known to be critical for receptor phosphorylation by GRKs and β-arrestin interaction. The real-time kinetic analysis using BRET showed a time-dependent increase in β-arrestin 2 recruitment with a plateau reached after 20–30 min of stimulation consistent with previous BRET data on β-arrestin recruitment to other GPCRs (26, 27, 33, 34, 51, 54). The sustained BRET signals induced after 20–50 min of stimulation suggests that hFSHR and hLHR present a class B GPCR profile similarly to the prototypic vasopressin V2 receptor (41, 46). In addition, the BRET data on β-arrestin 2 recruitment were nicely correlated with the internalization data in terms of efficiency and to some extent kinetics (Figure 7; Table 1). This is consistent with the previously reported central role played by β-arrestins in the internalization of hFSHR and hLHR (55, 56) and fits well with the classical paradigm of GPCR trafficking (41, 42, 57, 58). However, our real-time BRET analysis on both receptors clearly showed receptor recycling and/or recruitment of new receptors at the plasma membrane as indicated by a recovery of BRET signals occurring after 10 min of stimulation and reaching a maximum higher than the basal level after 45–60 min. Such an observation was specific to hFSHR and hLHR since the internalization of hV2R was significantly delayed with no recycling of the receptor observed, as previously reported for this receptor (48–50). This difference with V2R suggests that the trafficking of hFSHR and hLHR is more complex than their simple classification into class A versus B GPCRs. In fact, the recovery phase observed with hFSHR and hLHR may be explained either by the recycling of the internalized receptors as previously shown for FSHR (44, 59) and LHR (60), or by the mobilization of a new intracellular pool of “spare receptors” or “receptor reserve” to the plasma membrane, or a combination of both processes. Beside, both FSHR and LHR have been reported to traffic through pre-early endosomes (60). This unusual trafficking may explain the fast internalization and recycling observed in our system.

To get a better picture of what happens with hFSH and hLHR in terms of activation, desensitization, and internalization, we normalized the dose-dependent responses of both receptors with regard to cAMP pathway, β-arrestin 2 recruitment, and receptor internalization assessed by different BRET assays reported in this study (Figure 7). For both hFSHR (Figure 7A) and hLHR (Figure 7B), we found a spectacular left-warded shift (about three logs) of cAMP curve (EC_50_ ≈ pM) as compared to β-arrestin 2 and internalization curves (EC_50_ ≈ nM), indicating the high efficiency of gonadotropins for this signaling pathway. In contrast, there was no difference between β-arrestin 2 recruitment and internalization curves since both processes are tightly linked. Such a shift cannot be due to the fusion of the receptors with Rluc8 since both hFSHR-Rluc8 and hLHR-Rluc8 respond to gonadotropin stimulation with similar potencies as their respective unmodified receptors as shown by BRET (Figures 1D,E) and HTRF^®^ (Figures 1F,G) assays. In addition, dose–response curves of receptor internalization using BRET with KRas were similar to that for β-arrestin 2 recruitment even though no yPET-β-arrestin overexpression was used in the internalization assay, ruling out the possibility that the shift observed for β-arrestin 2 recruitment could reflect a diminished functionality of the yPET-β-arrestin 2 (Figures 5E and 7) or the modified variant in TANGO assay (Figure 5G).

Interestingly, the predicted receptor occupancy curves, using a *K*_d_ of 2 nM determined by radioligand binding assay on HEK 293 cells stably expressing hFSHR, indicated that <10% of the activated receptors is sufficient to promote maximal cAMP response whereas more than 90% of the receptors needed to be occupied to have full β-arrestin 2 recruitment as well as receptor internalization (Figures 7A,B). Noteworthy, our measurements of the rapid internalization phase displayed maximal response in the nanomolar range for both receptors, demonstrating that the full complement of receptor is accessible to hormone binding, even at early stimulation times. This observation rules out the scenario where only a limited fraction of receptors would be present at the plasma membrane at the time of stimulation. Together with the recycling data shown in Figure 6, our results are in accordance with the concept of “spare receptors,” postulating that for high-efficacy hormones, a small population of receptors occupied is sufficient to fully promote the biological response (61–63), a paradigm, which has also been previously evoked for gonadotropin receptors (61, 64–66). The “spare receptors” concept predicates that there is a mechanism by which only small amount of gonadotropin receptors needs to be occupied to fully elicit cAMP-dependent function of the gonadotropin hormones. This is consistent with the well-established amplification of the intracellular cAMP signaling pathway and suggests a model where Gαs and/or adenylyl cyclase would be limiting yet accessible to all the occupied receptors in the cells. Alternatively, the existence of pre-assembled receptor-G protein complexes, as demonstrated for many GPCR-G protein pairs (27, 51, 67–69), may explain such an observation. Indeed, a limited amount of pre-assembled complexes could preferentially bind hormones by virtue of its well-established affinity increase for the ligand within the ternary complex (70). In contrast, β-arrestin recruitment and receptor internalization processes are remarkably proportional to receptor occupancy, suggesting that neither mechanism is amplified but rather that they depend on a 1:1 stoichiometric interaction with the receptors. Moreover, the differences in hormone potencies and receptor efficacies between cAMP and β-arrestin 2 recruitment/internalization pathways may explain the balance between receptor activation and receptor desensitization but also the balance between G protein-dependent and β-arrestin-dependent signaling pathways. Therefore, further investigation will be required to better dissect these aspects of FSHR and LHR trafficking and their putative link with the G protein- and β-arrestin-dependent downstream signaling in the gonads in physiological and pathophysiological settings. From the technological point of view, our study illustrates the advantage of applying BRET and FRET approaches to study the signaling and trafficking of FSHR and LHR in real-time and live cells. Of course, these approaches are based on transient expression of fusion proteins of the receptors and their different signaling and regulatory partners. Therefore, it will be important in the future, to apply other methods in order to confirm our observations in cells or native tissues expressing unmodified receptors and regulatory proteins at endogenous levels. Despite these potential shortcomings, the assays presented here may nonetheless have considerable potential for pharmacological profiling of gonadotropin receptors.

## Conflict of Interest Statement

The authors declare that the research was conducted in the absence of any commercial or financial relationships that could be construed as a potential conflict of interest.
